# Trimetazidine in Heart Failure

**DOI:** 10.3389/fphar.2020.569132

**Published:** 2021-01-12

**Authors:** Hongyang Shu, Yizhong Peng, Weijian Hang, Ning Zhou, Dao Wen Wang

**Affiliations:** ^1^ Division of Cardiology, Department of Internal Medicine, Tongji Hospital, Tongji Medical College, Huazhong University of Science and Technology, Wuhan, China; ^2^ Hubei Key Laboratory of Genetics and Molecular Mechanism of Cardiologic Disorders, Huazhong University of Science and Technology, Wuhan, China; ^3^ Department of Orthopaedics, Union Hospital, Tongji Medical College, Huazhong University of Science and Technology, Wuhan, China

**Keywords:** trimetazidine, basic research, clinical research, non-ischemic heart failure, ischemic heart failure

## Abstract

Heart failure is a systemic syndrome caused by multiple pathological factors. Current treatments do not have satisfactory outcomes. Several basic studies have revealed the protective effect of trimetazidine on the heart, not only by metabolism modulation but also by relieving myocardial apoptosis, fibrosis, autophagy, and inflammation. Clinical studies have consistently indicated that trimetazidine acts as an adjunct to conventional treatments and improves the symptoms of heart failure. This review summarizes the basic pathological changes in the myocardium, with an emphasis on the alteration of cardiac metabolism in the development of heart failure. The clinical application of trimetazidine in heart failure and the mechanism of its protective effects on the myocardium are carefully discussed, as well as its main adverse effects. The intention of this review is to highlight this treatment as an effective alternative against heart failure and provide additional perspectives for future studies.

## Introduction

Cardiovascular disease is one of the most harmful diseases to human health, and heart failure represents the final clinical manifestation of all chronic cardiovascular diseases (Zhou et al., 2019). Although angiotensin converting enzyme inhibitors (ACEIs), angiotensin receptor antagonists (ARBs), beta-blockers, digitalis, spironolactone, furosemide, and other drugs are available, the prognosis of heart failure is still poor, as indicated by the low 5 years survival rate (Wenmeng and Qizhu, 2011). Therefore, heart failure research and the subsequent development of appropriate treatments still has a long way to go.

In Europe, trimetazidine has been used for the treatment of angina pectoris for more than 40 years. In 2000, trimetazidine was shown to directly improve myocardial metabolism by modulating beta oxidation, rather than by indirectly improving the hemodynamics (Kantor et al., 2000). In the past 20 years, clinical and basic studies on trimetazidine have confirmed its effectiveness in the treatment of heart failure. This article summarizes the basic pathophysiological processes of heart failure and discusses the clinical studies and associated molecular mechanism of trimetazidine in heart failure.

## Pathophysiological Process of Heart Failure

In heart failure, the cardiac contractile function decreases greatly, and the heart is unable to pump enough blood into the artery, resulting in ischemia and hypoxia, venous reflux obstruction, dyspnea, edema, and limb cyanosis. The burden of heart failure has increased to an estimated 23 million people. The incidence of heart failure for all ages is 2%–3% (3%–4% for people over 45 years old and 10% for people over 70 years old) (Vasan et al., 2018; Murphy et al., 2020).

A variety of external stimuli can promote the development of heart failure, including ischemia/hypoxia, pressure overload, and volume overload (Ziaeian and Fonarow, 2016). A series of compensatory changes occur in cardiomyocytes and non-cardiomyocytes when the myocardial tissue is exposed to stress. Most adult cardiomyocytes are terminally differentiated cells and possess very limited proliferation capacity (Zhu et al., 2020), while non-cardiomyocytes (fibroblasts, endothelial cells, inflammatory cells, and vascular smooth muscle cells) retain the ability to proliferate. Therefore, cardiomyocytes can only adapt to external changes through myocardial hypertrophy and metabolic remodeling, while other non-cardiomyocytes, including fibroblasts, proliferate in response to external stress, leading to structural changes in the heart.

### Cardiac Hypertrophy and Cell Death

Cardiac hypertrophy is characterized by an increased cross-sectional area of cardiomyocytes and enhanced protein synthesis, which is the most obvious and important change in myocardium under pressure or volume overload (Nakamura and Sadoshima, 2018). In a state of high pressure, the neuroendocrine system is activated, and vasoactive substances (angiotensin II, endothelin-I, vasopressin) are released into the blood. By binding to the corresponding receptors on the surface of cardiomyocytes, vasoactive substances activate the intracellular calmodulin kinase-nuclear factor of activated T cells (NFAT), mitogen-activated protein kinase (MAPK), and c-Jun N-terminal kinase (JNK) signaling pathways (Molkentin, 2013), and promote the expression of cardiac hypertrophy-related genes. Myocardial hypertrophy is an adaptive change of the myocardium in response to external pressure, which is conducive to the maintenance of basic systolic function in the compensation period. However, if external stimulation persists, cardiomyocytes die by apoptosis, necrosis, or autophagy-dependent cell death. The death of cardiomyocytes leads to a direct decrease in the number of cardiomyocytes, and it promotes hypertrophy of the remaining cardiomyocytes (Hariharan and Sussman, 2015). When the number of cardiomyocytes decreases by a certain extent, hypertrophic cardiomyocytes cannot fully compensate for cardiac function, leading to heart failure. Therefore, cardiomyocyte death is an important turning point from compensatory myocardial hypertrophy to heart failure.

### Myocardial Interstitial Fibrosis

In addition to alterations in cardiomyocytes, myocardial interstitial fibrosis is another important pathological change in heart failure (Lazzeroni et al., 2016). Myocardial interstitial fibrosis is characterized by a diffuse and disproportionate accumulation of collagen (collagen type I and III) in the myocardial interstitium, and it is known to aggravate heart failure in several ways (Gonzalez et al., 2018). First, the cross-linking of collagen directly reduces the diastolic function of the myocardium. Second, the rearrangement of collagen and cardiomyocytes indirectly reduces the force transfer between cardiomyocytes, which subsequently affect myocardial contractile function (Kasner et al., 2011). Furthermore, myocardial interstitial fibrosis induces ventricular arrhythmias during heart failure (Disertori et al., 2017). Studies have shown that myofibroblasts can act directly on cardiomyocytes during cell-cell contact, or can indirectly affect the sinoatrial node by secreting paracrine factors, affecting the normal rhythm and the downward transmission of rhythm, and ultimately leading to ventricular arrhythmias (Nguyen et al., 2017). Finally, myocardial interstitial fibrosis further aggravates the hypoxic symptoms of heart failure due to the increased oxygen diffusion distance (Kong et al., 2014).

### Changes in Myocardial Metabolism

The energy required by myocardial tissue is mainly provided by aerobic oxidation of fatty acids, supplemented by glucose, ketone bodies, amino acids, and lactic acid (Jansen, 2017). Although fatty acids serve as the main metabolic substrate, the aerobic oxidation efficiency of glucose is much higher, and glucose can maximize the production of ATP through the aerobic oxidation process. For example, 1 mole of 16-carbon fatty acid oxidation requires 46 moles of oxygen atoms and produces 105 moles of ATP, while the oxidation of 1 mole of glucose molecules requires only 12 moles of oxygen atoms, which produces 31 moles of ATP. Previous studies have shown that the myocardial ATP content decreases by 30%–40% during heart failure (Fukushima et al., 2015). To maintain adequate energy supply, the substrate for energy metabolism gradually changes from fatty acids to glucose. Moreover, it has been shown that patients with severe heart failure, such as advanced dilated cardiomyopathy, demonstrate lower expression of metabolic enzymes related to aerobic oxidation of fatty acids (long-chain acyl-CoA dehydrogenase and medium-chain acyl-CoA dehydrogenase). Peroxisome proliferator-activated receptors (PPARs) have also been shown to be significantly decreased during heart failure, resulting in a decrease in myocardial fatty acid utilization (Oka et al., 2012); in parallel, the expression of type I glucose transporter was upregulated and the utilization rate of glucose increased. The conversion of the energy metabolic substrate is beneficial for improving the efficiency of myocardial energy metabolism and alleviating the symptoms of heart failure. However, glucose glycolysis is more apparent during heart failure than during aerobic oxidation (Zhabyeyev et al., 2013), which results in excessive accumulation of protons and lactic acid in cardiomyocytes. Hydrogen ions are further exchanged with extracellular calcium ions through sodium-calcium channels, which leads to an overload of intracellular sodium and calcium (Fillmore and Lopaschuk, 2013). Therefore, most of the glucose acquired by cardiomyocytes is not used effectively; this not only reduces the efficiency of energy metabolism but also leads to calcium overload. Calcium overload can lead to endoplasmic reticulum stress (Nie et al., 2019; Mohsin et al., 2020) and mitochondrial dysfunction (Malyala et al., 2019), which can further aggravate myocardial injury.

## Basic Research of Trimetazidine in Heart Failure

### Energy Metabolism

**FIGURE 1:**
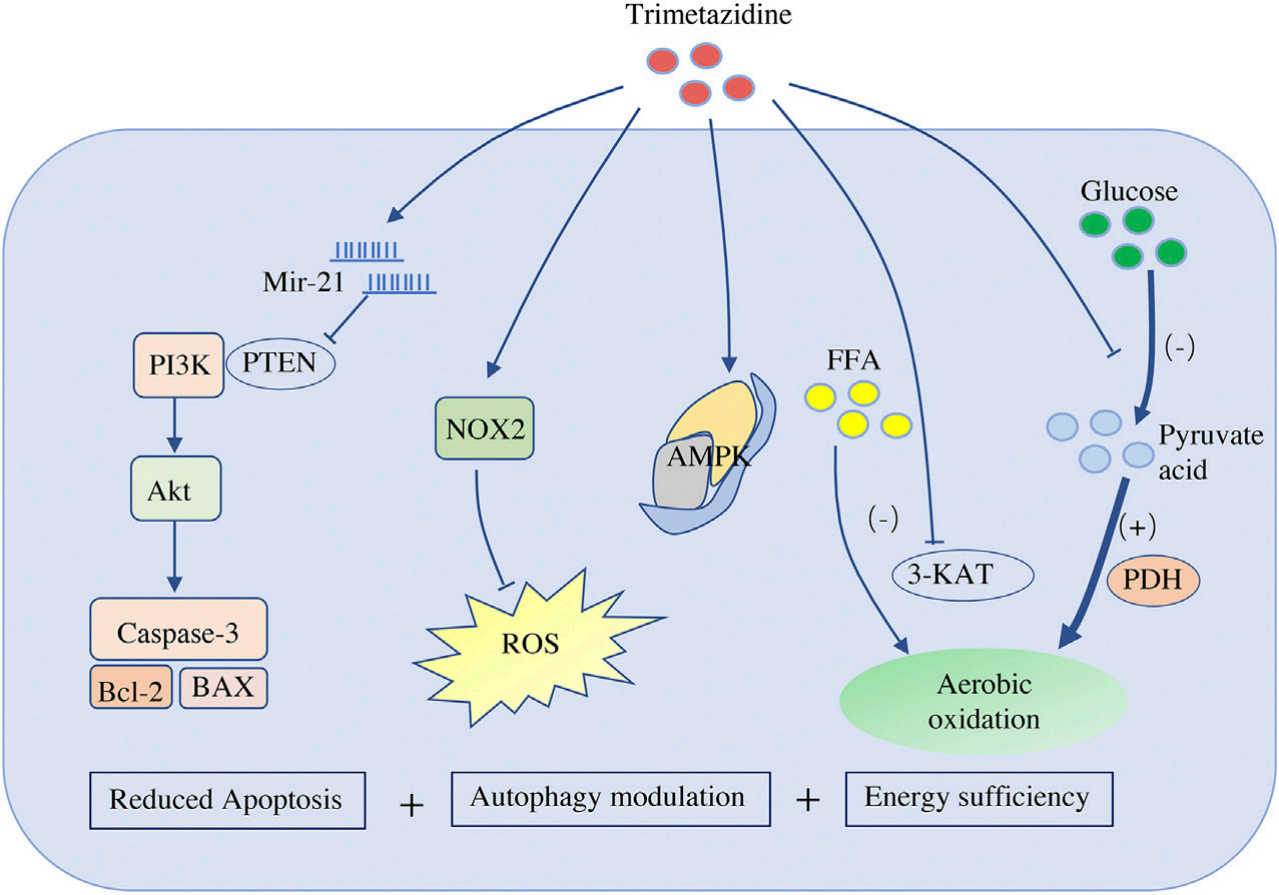
Effects of trimetazidine on cardiomyocytes. Trimetazidine promotes the production of mir-21, which targets and inhibits PTEN activity, and therefore activates PI3K-Akt signal pathway, and finally blocks the apoptosis pathway of cardiomyocytes by inhibiting the expression of Bax/Bcl-2 and caspase-3. Trimetazidine also promotes the expression of NADPH oxidase 2 and reduces ROS. It participates in the modulation of cardiomyocyte autophagy by regulating AMPK. Trimetazidine reduces the rate of aerobic oxidation of fatty acids by inhibiting the long-chain 3-ketoacyl-CoA thiolytic enzyme of mitochondria (a key enzyme for long-chain fatty acid beta oxidation), while indirectly increasing the activity of pyruvate dehydrogenase (the rate-limiting enzyme of aerobic oxidation of glucose). Therefore, it not only improves the efficiency of aerobic oxidation but also reduces the hydrogen ion produced by glycolysis. PTEN, phosphatase and tensin homolog; NOX2, NADPH oxidase 2; ROS, reactive oxygen species; AMPK, AMP kinase; FFA, free fatty acid; 3-KAT, 3-ketoacyl-CoA thiolytic enzyme; PDH, pyruvate dehydrogenase

More than 90% of the ATP in the heart is produced by aerobic oxidation. As mentioned above, in the case of heart failure, the energy supply is insufficient, so the heart preferentially chooses glucose, which leads to more efficient oxidation. Trimetazidine has been shown to reduce the rate of aerobic oxidation of fatty acids by inhibiting the mitochondrial long-chain 3-ketoacyl-CoA thiolytic enzyme, which is a key enzyme of long-chain fatty acid beta oxidation that catalyzes the last step of the fatty acid beta-oxidation cycle; this process indirectly increases the activity of pyruvate dehydrogenase, the rate-limiting enzyme of aerobic oxidation of glucose (Heggermont et al., 2016; Rosano and Vitale, 2018) (Figure 1). The evidence that administration of trimetazidine decreases the aerobic oxidation of fatty acids and encourages the utilization of glucose indicates that trimetazidine promotes the conversion of metabolic substrates and improves energy efficiency.

Glycolysis only takes place in the cytoplasm before glucose is fully oxidized in the mitochondria. Glycolysis produces pyruvate together with hydrogen ions; if excess pyruvate cannot successfully enter the mitochondria for oxidation, it will be reduced to lactic acid in the cytoplasm. During heart failure, glycolysis is enhanced, and consequently, hydrogen ions and lactic acid are increased. Indeed, previous clinical studies have shown that the level of plasma lactic acid in patients with heart failure is much higher than that in healthy people (Adamo et al., 2017). The level of lactic acid is closely related to the severity and prognosis of heart failure (Grodin and Tang, 2018); that is, a higher lactic acid content leads to worse prognosis (Zymliński et al., 2018; Biegus et al., 2019). Trimetazidine has been shown to alleviate lactic acidemia in right heart failure, and consequently promote the recovery of right ventricular function (Fang et al., 2012). In a rat model of cardiac ischemia, trimetazidine was shown to decrease the rate of myocardial glycolysis, enhance the aerobic oxidation of glucose, and promote post-ischemic repair (Kukes et al., 2013). The inhibition of glycolysis by trimetazidine effectively reduces the accumulation of hydrogen ions and lactic acid in the cytoplasm, thus avoiding adverse cardiac events, such as calcium overload (Swietach et al., 2013).

Taken together, the positive effects of trimetazidine on energy metabolism for heart failure is three-fold: First, it reduces the fatty acid metabolism by inhibiting the enzyme long-chain 3-ketoacyl-CoA thiolytic; second, it increases glucose metabolism by increasing the rate-limiting enzyme activity of glucose aerobic oxidation; and third, it inhibits excessive glycolysis and reduces the levels of hydrogen ions and lactic acid in the cytoplasm.

### Apoptosis of Cardiomyocytes

Cardiomyocyte apoptosis is a key factor that determines the transformation from compensation to decompensation. In a model of coronary artery microembolization-induced heart failure, trimetazidine significantly prevented cardiomyocyte apoptosis (Liu et al., 2015). In addition, similar to N-acetylcysteine (NAC), trimetazidine alone significantly reduced serum malondialdehyde (MDA) levels, infarct area, and apoptotic activity induced by ischemia-reperfusion, compared to those observed in the saline group (Senturk et al., 2014). Other studies have confirmed that trimetazidine antagonizes myocardial apoptosis by reducing the production of reactive oxygen species (ROS) and the expression of reduced form of nicotinamide-adenine dinucleotide phosphate (NADPH) oxidase 2 (Zheng and Liu, 2019) (Figure 1). Micro-RNA is also involved in the anti-apoptotic effect of trimetazidine, and miR-21 is known to be upregulated by trimetazidine (Liu et al., 2012; Yang et al., 2015). After targeting PTEN, miR-21 activates the PI3K-Akt signaling pathway and inhibits the expression of Bax/Bcl-2 and caspase-3, thus blocking cardiomyocyte apoptosis (Ma et al., 2016) (Figure 1).

### Myocardial Autophagy

Autophagy degrades longevity proteins and damaged or excessive organelles through lysosome-mediated pathways (Mialet-Perez and Vindis, 2017). Trimetazidine has been shown to enhance cardiac function by modulating cardiomyocyte autophagy. Indeed, administration of trimetazidine in diabetic cardiomyopathy rats was shown to enhance cardiomyocyte autophagy and improve cardiac function (Zhang et al., 2016). In addition, by promoting autophagy, trimetazidine also reduces sunitinib-induced cardiotoxicity in mice (Yang et al., 2019b). However, autophagy is a double-edged sword in myocardial tissue depending on the type and duration of stress. Moderate autophagy helps cardiomyocytes to survive and maintain the normal function of the heart, while excessive activation of autophagy may lead to decreased cardiac function and even heart failure (Ghosh and Pattison, 2018). In the ischemia-reperfusion injury model, moderate autophagy has a positive effect on cell recovery in the transient myocardial ischemic period. However, during the perfusion period, autophagy is strengthened, leading to excessive autophagy. This cytotoxic effect promotes excessive degradation and self-digestion of important components of cells, which causes irreversible damage to cardiomyocytes and eventually cell death. Therefore, inducing moderate autophagy and inhibiting excessive autophagy both contribute to the functional recovery of cardiomyocytes. Trimetazidine bidirectionally regulates cardiomyocyte autophagy during ischemia-reperfusion injury. In a hypoxia-reoxygenation injury model, in which trimetazidine induced moderate autophagy, trimetazidine alleviated cardiomyocyte damage by promoting AMPK autophagy influx (Zhong et al., 2017) (Figure 1). Furthermore, in an excessive autophagy model, which results from ischemia-reperfusion injury *in vivo*, the heart benefits from the inhibition of excessive autophagy following the activation of the Akt/mTOR signaling pathway by trimetazidine (Wu et al., 2018).

### Myocardial Interstitial Fibrosis

Connective tissue growth factor (CTGF) is a key molecule in myocardial interstitial fibrosis, which induces fibroblasts to proliferate and secrete extracellular matrix (Ramazani et al., 2018). Interestingly, trimetazidine effectively inhibits the expression of CTGF and reduces the accumulation of collagen I and collagen III in myocardial tissue (Zhang et al., 2020). The combination of irvabradine hydrochloride and trimetazidine has been shown to significantly reduce myocardial interstitial fibrosis caused by cardiac pressure overload (Ma et al., 2019). In an animal model of heart failure produced by transverse aortic constriction surgery, the expression of CTGF in rats treated with trimetazidine decreased by 34% compared to that in the normal saline group. ROS, which are negatively regulated by trimetazidine, induce the synthesis of CTGF (Zhao et al., 2019). Trimetazidine can improve the activity of NADPH oxidase and reduce the expression of ROS by regulating the translocation of the Rac1 subunit of NAPDH oxidase (Liu et al., 2010). Thus, trimetazidine may inhibit CTGF synthesis by reducing ROS production, which may improve myocardial interstitial fibrosis.

The improvement of myocardial interstitial fibrosis increased the efficiency of oxygen utilization by cardiomyocytes, alleviates the impaired contractility and compliance of the myocardium, and improves the symptoms of heart failure. The function of trimetazidine in myocardial fibrosis suggests that the protective effects of trimetazidine on the heart may not only depend on cardiomyocytes; therefore, the effects on non-cardiomyocytes and extracellular remodeling need to be explored further.

### Myocardial Inflammation

Heart failure evokes inflammation, and TNF-α, IL-6, IL-18, and atrial natriuretic peptide (ANP) can be induced in pressure overload-induced heart failure. In contrast, the release of cardiac cytokines deteriorate heart function and induce heart failure (Shirazi et al., 2017; Van Linthout and Tschöpe, 2017). Trimetazidine has anti-inflammatory effects and has been shown to significantly decrease the levels of serum inflammatory markers (IL-1 β, IL-6, and TNF-α) (Zhou et al., 2012). In the model of coronary artery microembolism in Guangzhou Bama miniature pigs, inflammatory markers, such as PDCD4, NF-kB, and TNF-α, were shown to increase by 4–8 fold (Su et al., 2017) and were shown to decrease by about 40% following treatment with trimetazidine. Trimetazidine combined with coenzyme Q10 is effective against viral myocarditis (Shao et al., 2016) and alleviates septic myocardial damage induced by endotoxin by activating sirt1 (Chen et al., 2016) and promoting the migration of central granulocytes (Chen et al., 2018).

## Clinical Studies of Trimetazidine in Heart Failure

The recommendation for the utilization of trimetazidine in stable coronary syndrome is at the IIa level (Knuuti et al., 2020). The 2016 European Society of Cardiology (ESC) guidelines on heart failure recommended TMZ for the relief of persistent angina pectoris in patients in combination with a beta-blocker or an alternative agent at the IIb level (Ponikowski et al., 2016). Many randomized controlled trials have confirmed the protective effects of trimetazidine in heart failure by improving clinical manifestations and cardiac function (Fragasso et al., 2006a; Fragasso et al., 2011; Grajek and Michalak, 2015). In terms of mechanisms, trimetazidine has been shown to reduce the expression of atrial natriuretic peptide (ANP) (Morgan et al., 2006), increase left ventricular high-energy phosphate levels (Fragasso et al., 2006b), and reduce the risk of arrhythmias in heart failure (Gunes et al., 2009b; Cera et al., 2010). Furthermore, trimetazidine can improve the symptoms of heart failure by reducing the energy consumption of the whole body.

### Chronic Ischemic Heart Failure

Ischemic heart disease is an important cause of heart failure, and clinical studies have also shown that trimetazidine has a significant protective effect against chronic ischemic heart failure. A meta-analysis (including 17 clinical trials, 955 patients) showed that trimetazidine dramatically increased the ejection fraction of patients with heart failure (weighted mean difference [WMD], 7.49%; 95% confidence interval [CI], 6.26 to 8.71; *p* < 0.01) (Gao et al., 2011). Furthermore, subgroup analysis showed that trimetazidine also had a positive effect on patients with ischemic heart failure (WMD, 7.37%; 95% CI, 6.05 to 8.70; *p* < 0.01). Table 1 shows the randomized controlled study of trimetazidine in heart failure from 2000 to 2020.

**TABLE 1:** Randomized controlled study of trimetazidine in heart failure.

year	study	patients (T/c)	NISH	ISH	LVEF	TMZ (mg/d)	Time	LVEF*	other endpoints	Positive/none
2004	Thrainsdottir et al. (2004)	10/9	50%	50%	<40%	60	1 month	33 ± 8% vs. 37 ± 16%	/	none
2006	Fragasso et al. (2006b)	12/12	/	/	<45%	60	90 days	34 ± 10% vs. 39 ± 10%	PCr / ATP: 1.80 ± 0.50 vs. 1.35 ± 0.33	none
2006	Fragasso et al. (2006a)	28/27	/	100%	<45%	60	13 months	43 ± 10% vs. 34 ± 7%	/	postive
2007	Di Napoli et al. (2007)	30/31	34.40%	100%	<35%	60	48 months	∼40% vs. ∼30%	/	positive
2008	Tuunanen et al. (2008)	12/7	/	100%	<40%	70	3 months	34.8 ± 12% vs. 31.9 ± 12%	/	positive
2009	Gunes et al. (2009a)	51/35	29%	66%	<40%	60	3 months	42.4% ± 6.3% vs. 33.2% ± 6.6%	/	positive
2009	Gunes et al. (2009b)	36/36	/	33%	<40%	60	6 months	32.7 ± 6.5% vs. 37.2 ± 5.5%	Max P-wave duration :106.7 ± 15.8 vs. 91.7 ± 12.7 ms	none
2010	Cera et al. (2010)	17/13	/	60%	<45%	60	6 months	40.11 ± 1.23% vs. 37.97 ± 13.21%;	QTc interval duration: 451.81 ± 55.02 vs. 453.20 ± 51.50	positive
2011	Fragasso et al. (2011)	25/19	34%	66%	<45%	60	36 months	42 ± 11% vs.36 ± 6%	REE: 1,580 ± 263 vs. 1,690 ± 337 kcal/day	positive
2014	Winter et al. (2014)	30/30	100%	0	<45%	70	6 months	31 ± 10% vs. 34 ± 8%	6MWT: 443 ±25 vs. 506 ±79 m; (18) FDG-PET SUV: 7.0 ±3.6 vs. 8.2±3.4	none
2016	Momen et al. (2016)	55/53	/	100%	<40%	70	6 months	36.6% vs. 31.2%	/	positive
2016	Jatain et al. (2016)	52/48	46%	/	<45%	60	3 months	30.9% vs 27%	6MWT: 402 vs. 349.7 m	positive

T/c, TMZ/control, trimetazdine group/control group; NISH, non ischemic heart disease; ISH, ischemic heart disease; LVEF, left ventricular ejection fraction stated in the inclusion criteria of the clinical trial, LVEF represents the fact that all patients included in the study had an LVEF < the number indicated; LVEF *, the average LVEF of all patients after treatment, trimetazdine group vs. control group; TMZ, trimetazidine; 6MWT, 6 min walk test; REE, Whole body resting energy expenditure; FDG-PET, β-2-[18 F]-Fluoro-2-deoxy-D-glucose-Positron Emission Tomography; PCr/ATP, phosphocreatine/adenosine triphosphate.

Previous clinical studies have shown that the treatment time for trimetazidine ranges from 1 to 48 months. The improvement of the ejection fraction (EF) value of the heart is not obvious in short-term treatment (≤6 months). A previous study of 19 patients with heart failure with EF values less than 40% who received trimetazidine for 1 month showed no significant difference in cardiac function compared to the placebo group (Thrainsdottir et al., 2004). Subsequently, Gunes et al. expanded the sample size to 60 people and prolonged the treatment time to 6 months; however, the trimetazidine group still showed no benefit at the end of the treatment (EF, trimetazidine vs. placebo, 32.7% ± −6.5% vs. 37.2% ± −5.5%) (Gunes et al., 2009b). In contrast, in the trial conducted by Tuunanen et al., there was a significant improvement in cardiac function (EF, trimetazidine vs. placebo, 34.8% ± 12% vs. 31.9% ± 12%) in patients with heart failure who received trimetazidine (70 mg per day) for only 3 months (Tuunanen et al., 2008). These differences may be due to differences in ultrasound testing and selection bias as a result of the small sample size.

Unlike short-term treatment, trimetazidine has been shown to improve heart function in chronic treatment for more than 1 year. In a trial of 55 people with heart failure with an EF value of less than 45%, the trimetazidine group showed a significant improvement in EF value compared to the placebo group (trimetazidine vs. placebo, 43% ± 10% vs. 34% ± 7%) after receiving trimetazidine 60 mg daily for 13 months (Fragasso et al., 2006a). Furthermore, the trimetazidine group showed a greater improvement in EF value (trimetazidine vs. placebo, 42% ± −11% vs. 36% ± −6%) when the follow-up time was extended to 36 months (Fragasso et al., 2011). Moreover, in a study by Di Napoli et al., the effect of trimetazidine was more significant (trimetazidine vs. placebo,∼40% vs. ∼30%) than in the placebo group, who had lower EF values (<35%) and longer treatment time (48 months); these findings indicate that the longer the treatment time of trimetazidine, the more significant the improvement of cardiac function.

However, it is worth noting that the population samples included in the current clinical studies on trimetazidine are very small, and there is a probability of overestimating the clinical benefit. Second, the follow-up time was relatively short; only one study lasted 48 months, and most lasted 3–6 months. In addition, the side effects of trimetazidine have not been thoroughly evaluated. Therefore, large-scale, prospectively designed, randomized, double-blinded trials are still required to verify the cardiac benefits of trimetazidine and evaluate its adverse effects (Gao et al., 2011).

In addition to improving the EF value, trimetazidine can also improve arrhythmia by reducing heart rate variability and shortening the QTc interval (Gunes et al., 2009a; Zemljic et al., 2010). In addition, trimetazidine has been shown to improve endothelium-dependent relaxation (EDR), which correlates with improved nitric oxide (NO) bioavailability and reduced ROS levels (Godo and Shimokawa, 2017). Wu et al. proved that superoxide dismutase (SOD) activity, eNOS expression, and the production of NO were all elevated in endothelial progenitor cells pretreated with trimetazidine (Wu et al., 2013). Moreover, trimetazdine also significantly reduced plasma brain natriuretic peptide (BNP) and cardiac troponin T levels in patients with ischemic heart failure (Di Napoli et al., 2007; Rehberger-Likozar and Šebeštjen, 2015). Long-term use of trimetazidine has also been shown to be effective at increasing exercise endurance (Sisakian et al., 2007) and reducing all-cause mortality (Grajek and Michalak, 2015). With regards to patients with ischemic heart failure and diabetes (Fragasso et al., 2003), trimetazidine administration was shown to improve liver function (Li et al., 2017) and lower blood glucose and myocardial glucose metabolism. In addition, the therapeutic benefits of trimetazidine have been shown to be increased when combined with other non-heart failure first-line medicines. For example, trimetazidine combined with bisoprolol significantly increased the ventricular ejection fraction in patients with chronic heart failure compared to bisoprolol alone (Ke et al., 2016). Moreover, treatment with trimetazidine and Shexiang Baoxin pills improved the clinical symptoms of ischemic heart failure to a greater extent than with Shexiang Baoxin pills alone (Wen et al., 2018).

Previous studies have shown that trimetazidine improves cardiac function by improving hemodynamics; however, the protective effect of trimetazidine on ischemic heart failure is not entirely due to its effects on the hemodynamic system. Indeed, trimetazidine has been shown to significantly reduce the number of angina pectoris attacks per week in patients with heart failure, without changing the ejection fraction (3.9 vs. 5.7, *p* < 0.01), as well as significantly improve the exercise ability of patients (6 min walking test, trimetazidine vs. placebo, 245 m vs. 210 m, *p* < 0.05) (Sisakian et al., 2007). Furthermore, trimetazidine can alleviate skeletal muscle damage caused by statins and promote functional recovery (Song et al., 2018). Trimetazidine has also been shown to help to mitigate the damage to lung function caused by high altitude reactions and hypoxia at high altitudes (Yang et al., 2019a). In addition, trimetazidine can reduce renal ischemia-reperfusion injury by reducing the expression of erythroid-derived nuclear factor-2 related factors (Amini et al., 2019). Therefore, it is speculated that the protective effect of trimetazidine on ischemic heart failure not only comes from the direct effect on myocardial tissue but also the indirect effect on other tissues and organs.

### Non-Ischemic Heart Failure

Trimetazidine also has a protective effect against non-ischemic heart failure, as shown by a meta-analysis by Gao et al. (WMD: 8.72%; 95% CI:5–51 to 11.92; *p* < 0.01) (Gao et al., 2011; Tarkin and Kaski, 2018). In a randomized parallel study conducted in patients with diabetic cardiomyopathy over the course of 1 month, trimetazidine elevated the ejection fraction by approximately 5% compared to that observed in the placebo group (Rosano et al., 2003). Therefore, early application of trimetazidine has the potential to prevent the occurrence or ameliorate the degree of diabetic cardiomyopathy and reduce the incidence of heart failure caused by diabetic cardiomyopathy (Wenmeng and Qizhu, 2011). One explanation for this observation is that trimetazidine optimizes the substrate metabolism of dilated cardiomyopathy (Jatain et al., 2016) and improves systemic insulin sensitivity (Zhang et al., 2016). However, one previous study reported that there was no significant difference in ejection fraction, exercise tolerance, or quality of life between trimetazidine (70 mg/day for 6 months) and placebo for non-ischemic heart failure Winter et al., (2014). One potential reason for this may be that trimetazidine is more effective in patients with fatty acid oxidative disorders, such as diabetes and obese individuals (Ussher et al., 2014; Tang et al., 2019), while the prevalence of diabetes in this cohort was only 8%. Non-ischemic heart failure has a variety of causes, including diabetes and hypertension; therefore, specific treatments targeting the causes may provide more promising outcomes than simply relieving the symptoms of heart failure. In view of this, a more detailed subgroup analysis of different causes of heart failure is needed in order to obtain more accurate treatment.

### Adverse Effects

Clinical pharmacological studies suggest that although common adverse reactions of trimetazidine can be found occasionally, such as gastrointestinal discomfort, nausea and vomiting, and rarely reported thrombocytopenia, agranulocytosis, and liver dysfunction (Chrusciel et al., 2014), these adverse reactions tend to disappear after withdrawal (Meiszterics et al., 2017). In the past, there was a concern that trimetazidine might be linked to Parkinson-like syndrome. A report that eight patients developed Parkinson’s disease while taking trimetazidine was first published in 2004 (Martí Massó, 2004). Then, several cases of Parkinson’s disease induced by trimetazidine were reported in the following years (Masmoudi et al., 2012; Kwon et al., 2019; Pintér et al., 2019; Pintér et al., (2020); Dy et al., 2020). However, all these reported Parkinson’s disease were studies based on a small series of patients and case reports. A recent published trial focusing on efficacy and safety of trimetazidine after percutaneous coronary intervention (ATPCI), which recruited 6,007 patients with a median follow-up of 47.5 months, showed that the occurrence of neurological symptoms such as Parkinson’s disease or drug induced parkinsonism were similar in the placebo and trimetazidine arms, which provided strong evidences that trimetazidine had no association with those neurological symptoms. In addition, this trail also showed there were no statistically significant differences in thrombocutopenia, agranulocytosis, hepatic disorders, etc. between treatment groups (Ferrari et al., 2020).Therefore, trimetazidine is relatively safe even in long-term prescription.

## Summary and Outlook

Until now, heart failure has been a huge challenge for cardiovascular doctors. Myocardial hypertrophy, apoptosis, myocardial interstitial fibrosis, and myocardial metabolic remodeling are all important pathological factors leading to heart failure. There are a variety of medicines used for the treatment of heart failure, among them, trimetazidine has a unique position in cardiovascular therapy because of its ability to optimize energy metabolism (Marzilli et al., 2019). Trimetazidine has been recommended for the treatment of stable coronary artery diseases since 2013, but it has not been recommended for heart failure (Montalescot et al., 2013; Milinković et al., 2016). Although a number of double-blind controlled trials have confirmed the effectiveness of trimetazidine in the treatment of heart failure, the total number of cases included is limited, and large-scale multicenter clinical studies are still required.

Many basic studies have confirmed that trimetazidine has positive effects, including against myocardial fibrosis and apoptosis, as well as anti-inflammatory effects. Double-blind controlled trials have demonstrated the effectiveness of trimetazidine in ischemic and diabetic heart failure, but whether trimetazidine has a good protective effect on other types of heart failure with decreased ejection fractions remains unknown. In addition, the effect of trimetazidine on heart failure is not limited to cardiomyocytes, and clinical studies have found that trimetazidine also improves EDR (Belardinelli et al., 2007) and reduces inflammation (Shao et al., 2016) in heart failure. Basic studies have also shown the protective effects of trimetazidine on non-cardiomyocytes, including antifibrotic effects (Zhao et al., 2019). Therefore, exploring the effect of trimetazidine on non-cardiomyocytes will be important in future studies.

Heart failure with preserved ejection fraction accounts for a large proportion of patients with heart failure (Dunlay et al., 2017), and its inducing factors, development, and changes in internal energy metabolism are quite different from those of heart failure with decreased ejection fraction (Borlaug, 2014; Redfield, 2016). Given that trimetazidine exhibits many beneficial effects on the cardiovascular system, it is considered likely that it could also prove useful in the treatment of ejection fraction preserved heart failure. However, there have been no clinical studies of trimetazidine on preserved ejection fraction heart failure. An ongoing randomized double-blind controlled trial of trimetazidine in ejection fraction preserved heart failure may provide some preliminary answers as to whether trimetazidine is effective in these patients (van de Bovenkamp et al., 2020). Moreover, there is no basic research on trimetazidine in preserved ejection fraction heart failure, and more in-depth research is required. Research in this field is lacking and immature, and further studies of the use of trimetazidine in heart failure with preserved ejection fraction may bring considerable benefits, both medically and economically.

## Author Contributions

HS designed and drafted the manuscripts; YP, NZ, WH, and DW critically revised the entire manuscript; and all authors approved the entire submitted and final versions. We would like to thank Editage (www.editage.cn) for English language editing.

## Funding

This work was supported by the National Natural Science Foundation of China (No. 81570261).

## Conflict of Interest

The authors declare that the research was conducted in the absence of any commercial or financial relationships that could be construed as a potential conflict of interest.
